# Metagenomic profiling of the gut microbiome to predict orthopedic healing responses in postmenopausal women

**DOI:** 10.3389/fcimb.2026.1771312

**Published:** 2026-02-09

**Authors:** Hongyi Pan, Lianguo Wu, Shaoqin Sheng

**Affiliations:** 1 Second Clinical Medical College, Zhejiang Chinese Medical University, Hangzhou, Zhejiang, China; 2 Department of Orthopedics II, Second Affiliated Hospital of Zhejiang Chinese Medical University, Hangzhou, Zhejiang, China; 3 Department of Gynecology, Hangzhou Women’s Hospital (Hangzhou Maternity and Child Health Care Hospital), Hangzhou, Zhejiang, China

**Keywords:** gut microbiome, microbial diversity, orthopedic healing, personalized medicine, postmenopausal women, predictive biomarkers, tissue repair

## Abstract

**Introduction:**

Recovery following orthopedic procedures in postmenopausal women is often prolonged and more complex due to age-related physiological changes, including reduced bone mineral density, altered hormonal profiles, impaired immune regulation, and delayed tissue regeneration. Conventional recovery assessment methods such as radiographic imaging, range-of-motion evaluation, and functional mobility tests provide valuable clinical information but offer limited insight into the underlying biological processes that influence healing. Emerging evidence indicates that the gut microbiome plays a critical role in regulating inflammation, immune homeostasis, and tissue repair, highlighting its potential as a predictive biomarker for post-surgical recovery outcomes. This study investigated the association between gut microbiome dynamics and recovery following orthopedic surgery in postmenopausal women.

**Methods:**

Stool samples were collected from preoperative (baseline) and 6 weeks postoperative time points. Microbial profiling was performed using 16S rRNA gene sequencing on the Illumina MiSeq platform, and data processing and taxonomic analysis were conducted using QIIME2. Microbial diversity was evaluated through alpha diversity metrics to assess community richness and beta diversity to characterize compositional differences over time. Clinical recovery was assessed using radiographic imaging, the Western Ontario and McMaster Universities Osteoarthritis Index (WOMAC), and the Timed Up and Go (TUG) functional mobility test. To evaluate the predictive potential of the gut microbiome, a random forest machine learning model was trained using microbial abundance data and correlated with clinical recovery outcomes.

**Results:**

The results revealed significant temporal shifts in gut microbial composition during the recovery period. Bacterial diversity varied across time points, with Firmicutes and Bacteroidetes identified as the dominant phyla. Increased abundance of these taxa was strongly associated with improved functional outcomes and faster recovery. In contrast, elevated levels of Proteobacteria and Escherichia were linked to delayed healing and poorer clinical performance. The predictive model achieved an accuracy of 85%, demonstrating the robustness of gut microbiome signatures as indicators of postoperative recovery.

**Discussion:**

Overall, this study highlights the significant influence of gut microbiome composition on orthopedic recovery in postmenopausal women. Identification of microbial biomarkers associated with favorable healing outcomes provides a foundation for developing microbiome-guided, personalized therapeutic strategies to enhance postoperative recovery and improve long-term musculoskeletal health.

## 1 Introduction

Age-related physiological changes such as decreased bone mineral density, hormonal imbalance, and delayed tissue regeneration often impact orthopedic recovery in postmenopausal women (Kamel et al., 2022). These elements may raise the risk of poor bone healing, delayed union, and general difficulties with healing. Standard clinical instruments like radiographic imaging, physical examinations, and mobility assessments are still essential for tracking advancement (Meylani, 2024), but they don’t accurately capture the underlying biological variability that influences each person’s healing response. Finding new biomarkers that can enhance recovery outcome prediction and direct focused rehabilitation tactics is therefore of increasing interest (Cullen et al., 2021).

Understanding how the gut microbiota affects more general aspects of human health has received more attention in recent years (Watson et al., 2023). Several vital processes, such as immune modulation, inflammation control, and tissue repair, are supported by the gut microbial community, which is made up of a variety of bacteria and other microorganisms (Colella et al., 2023). Recent research indicates that bone physiology, gut microbiota, and post-surgical recovery are closely related. Short-chain fatty acids (SCFAs), one type of microbial metabolite, are essential for controlling immunological response, systemic inflammation, and bone remodeling (Eshghjoo et al., 2021). These connections suggest that gut microbiome profiles could be useful indicators of healing after orthopaedic treatments (Sokol et al., 2022).

Using this framework as a foundation, our research examines whether gut microbial patterns can be used to predict orthopedic recovery in postmenopausal women (Nadeem-Tariq et al., 2025). We suggest that differences in microbial composition affect immune-related pathways and inflammation, both of which are essential for the healing process (Leibovitzh et al., 2022). In order to investigate this theory, we used high-throughput sequencing and PCR amplification of the 16S rRNA gene to examine the gut microbiome at two points in time: preoperative (baseline) and 6 weeks postoperative. As recovery proceeds, these time points aid in capturing microbiome dynamics (Dreier et al., 2022).

In addition to microbial profiling, thorough clinical assessments were carried out to evaluate postoperative function and bone healing. Callus growth and bone regeneration were monitored using radiographic imaging (Liu et al., 2022). Standardized instruments that assess patient-reported symptoms and mobility performance, such as the Timed Up and Go (TUG) test and the WOMAC index, were used to measure functional recovery (Ferreira et al., 2022. Finding microbial taxa linked to successful healing outcomes is made easier by combining these clinical metrics with microbiome data, which offers a more comprehensive understanding of recovery patterns (Wang et al., 2022).

Machine learning techniques, especially random forest classifiers, were used to assess whether particular microbial characteristics could accurately predict recovery responses in order to increase prediction accuracy (Freitas et al., 2023). Microbial groups that contribute most significantly to these predictions were further highlighted by feature importance analyses (Ancira et al., 2025). These discoveries could facilitate the creation of microbiome-based biomarkers in the future, allowing orthopedic patients to receive individualized recovery plans (Nadeem-Tariq et al., 2025).

**Figure 1:**
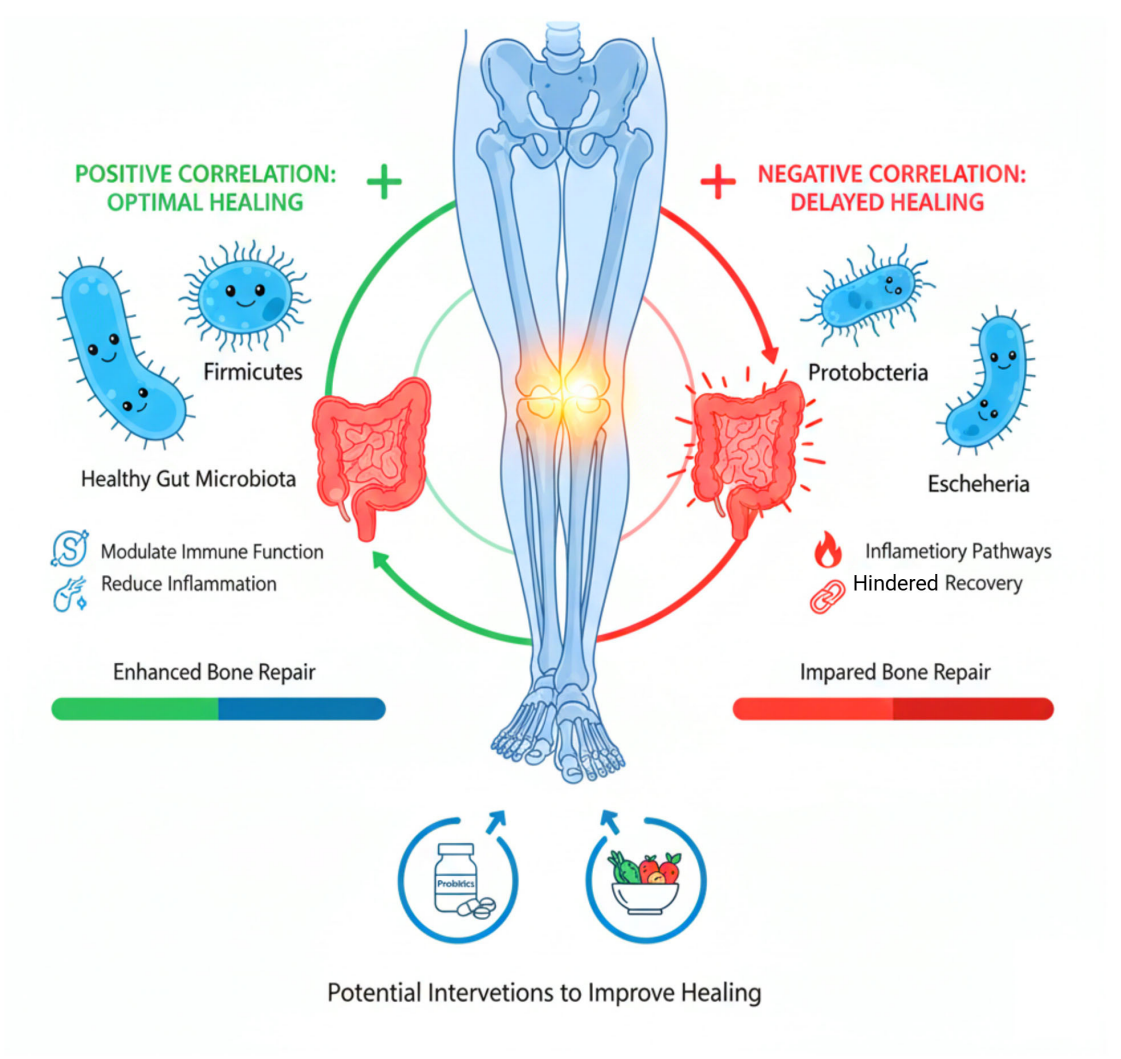
Microbiome-bone healing axis in postmenopausal women.

The overall goal of this study is to investigate how gut microbial communities affect surgical recovery in postmenopausal women in order to connect microbiome science with orthopedic rehabilitation. Finding microbial taxa that are associated with healing may lead to targeted therapeutic strategies to improve postoperative outcomes, such as nutritional interventions, microbiome-modulating treatments, or customized probiotics. This research mainly highlights the significance of the gut microbiome in influencing orthopedic recovery and adds to the growing field of microbiome-based precision medicine. **Figure 1** depicts the overall study design.

## 2 Materials and methods

### 2.1 *In vitro* methods

#### 2.1.1 Participants and sample collection

This study included postmenopausal women undergoing elective orthopedic surgery. The inclusion and exclusion criteria for participant selection, as well as their demographic characteristics, are summarized in **Table 1**. Key perioperative variables, such as antibiotic use, analgesia regimen, and nutritional status, were carefully recorded, as they may influence both microbiome composition and the healing process. The sample size of 50 participants was chosen based on practical considerations, including the availability of participants and resources for microbiome profiling and clinical assessments. Postmenopausal women undergoing orthopedic surgery had their feces collected at two crucial intervals: preoperative (baseline) and 6 weeks postoperative. In order to preserve the stability of microbial DNA, participants’ stool samples were quickly stored at –80°C after being placed in sterile collection containers. The genetic material was preserved for use in subsequent microbiome analyses thanks to this preservation technique. By taking samples at these two intervals, we were able to track changes in the composition of gut microbes during the postmenopausal patients’ recuperation.

**Table 1:** Participant demographics.

Category	Details
Inclusion Criteria	1. Postmenopausal women (aged 50–70 years)
	2. Undergoing elective orthopedic surgery
	3. Written informed consent provided
	4. No prior gastrointestinal disorders affecting microbiome
Exclusion Criteria	1. Chronic antibiotic use within 3 months before surgery
	2. History of gastrointestinal diseases (e.g., IBD, Crohn’s disease)
	3. Severe renal, liver, or immune dysfunction
	4. Use of probiotics or prebiotics within 1 month prior to the study
Total Number of Participants	50
Demographic Characteristics	1. Age: 55 ± 7 years
	2. BMI: 26.5 ± 4.1 kg/m²
Types of Orthopedic Surgeries Performed	1. Total knee arthroplasty (TKA)
	2. Hip replacement surgery (total or partial)
	3. Fracture fixation surgeries
Key Perioperative Variables	1. Antibiotic use: Standard prophylactic antibiotic regimen
	2. Analgesia regimen: Combination of opioids and NSAIDs
	3. Nutritional status: Preoperative malnutrition risk assessed by SGA
Potential Confounders	1. Smoking status (active/smoker/non-smoker)
	2. Preoperative comorbidities (e.g., diabetes, hypertension)

#### 2.1.2 DNA extraction

The QIAamp DNA Stool Extraction Kit (Qiagen USA), which is designed for the effective recovery of high-quality DNA from stool material, was used in this study in order to isolate genomic DNA from each fecal sample. To guarantee efficient inhibitor removal while preserving DNA integrity, every extraction step was carried out in accordance with the manufacturer’s instructions. Using a NanoDrop spectrophotometer (Thermo Fisher Scientific, USA), the extracted DNA’s concentration and purity were evaluated, ensuring that the samples had satisfied the specifications for subsequent PCR amplification and sequencing.

#### 2.1.3 16S rRNA gene amplification

The V3–V4 region of the 16S rRNA gene was amplified using the forward primer 5-CCTACGGGNGGCWGCAG-3 and the reverse primer 5-GGACTACHVGGGTATCTAAT-3 in order to characterize the intestinal microbiota. Because of its strong discriminatory power, this region is frequently used for bacterial taxonomy. Denaturation at 98°C for two minutes was the first step in the PCR protocol. This was followed by thirty cycles of amplification, each lasting ten seconds at 98°C, thirty seconds at 55°C, and thirty seconds at 72°C. A final extension step was carried out for five minutes at 72°C. To ensure that only pure high-quality amplicons were sent forward for sequencing, amplified products were purified using Agencourt AMPure XP beads (Beckman Coulter, USA), to remove primer dimers and non-specific fragments.

#### 2.1.4 Sequencing

The Illumina MiSeq platform (Illumina USA), which offers high-throughput high-accuracy sequencing appropriate for microbial community studies, was used to sequence the purified amplicons. The sequencing depth was targeted at (specify average sequencing depth, e.g., 50, 000 reads per sample) to ensure sufficient coverage of the microbiome. The average number of high-quality sequences retained after quality control was (specify number, e.g., 45, 000 sequences per sample). By directly comparing the microbial composition before and after surgery, sequencing outputs had made it possible to identify changes related to orthopedic recovery.

#### 2.1.5 Microbial community profiling

**Figure 2:**
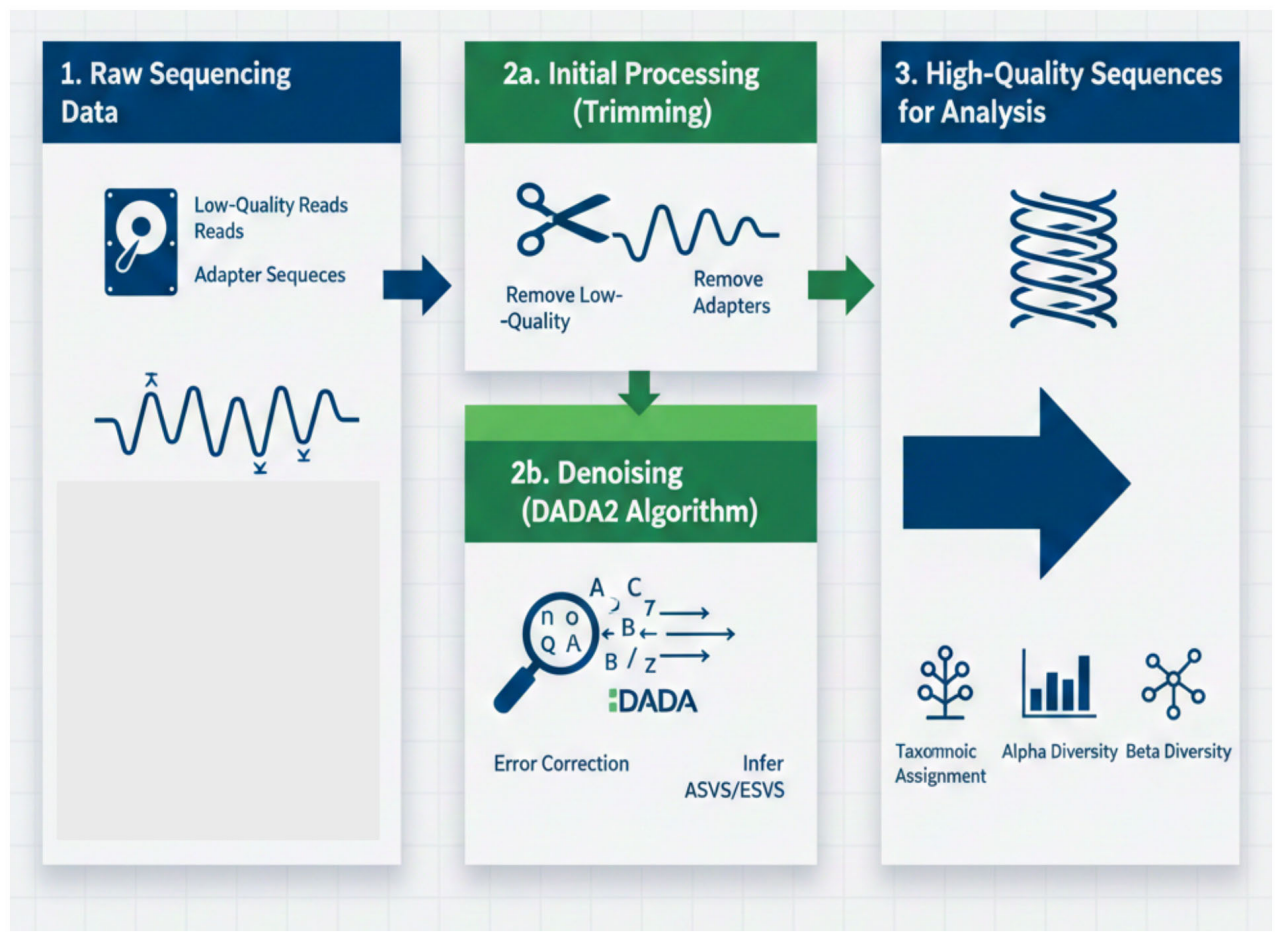
Microbiome data processing workflow.

The QIIME2 pipeline (v2021.2) was used to analyze the sequencing data. First, low-quality reads were eliminated, and sequencing errors were fixed through quality filtering. DADA2 was used for denoising, guaranteeing that only high-fidelity sequences were kept. Operational taxonomic units (OTUs) were created from the processed sequences by applying a 97 percent similarity threshold. The SILVA reference database (v138) was used for taxonomic assignment, which allowed the identification of the bacterial taxa found in each sample. In order to compare community differences between the two time points and assess microbial richness within each sample, both alpha and beta diversity metrics were computed. **Figure 2** shows the entire workflow for data processing.

#### 2.1.6 Healing assessment

A combination of functional mobility tests and radiographic imaging was used to assess the participants ‘ recovery. X-ray scans were used to record structural changes over time and track bone healing. Standardized instruments such as the Timed Up and Go (TUG) test and the WOMAC index, which measure pain, stiffness, and mobility after surgery, were used to gauge functional improvements. These assessments were carried out at baseline and once more six weeks following surgery, allowing participants to be categorized according to their clinical progress into fast-healing and slow-healing groups.

Radiographic Healing (Callus Formation & Bone Alignment)

- Fast-Healing: At least 50% callus formation and clear bone alignment at six-week follow-up (based on radiographic imaging).
- Slow-Healing: Less than 50% callus formation or evidence of delayed union or malalignment.

Percentage Improvement in WOMAC Scores (Pain, Stiffness, Function)

- Fast-Healing: A minimum of 30% improvement in the total WOMAC score from baseline at six weeks.
- Slow-Healing: Less than 20% improvement in the total WOMAC score from baseline at six weeks.

Timed Up and Go (TUG) Test Time (Functional Mobility)

- Fast-Healing: A reduction of at least 20% in TUG test time from baseline at six weeks.
- Slow-Healing: A reduction of less than 10% in TUG test time or no improvement.

Composite Classification

- Participants were classified as “Fast-Healing” if they met at least two out of three of the following criteria:
  - At least 50% callus formation and good alignment in radiographic imaging.
  - A 30% improvement in WOMAC score.
  - A 20% or greater reduction in TUG test time.
- Participants were classified as “Slow-Healing” if they met at least two out of three of the following criteria:
  - Less than 50% callus formation or poor alignment.
  - Less than 20% improvement in WOMAC score.
  - Less than a 10% reduction in TUG test time.

### 2.2 In silico methods

#### 2.2.1 Differential abundance analysis

Using the DESeq2 package, differential abundance analysis was performed to find microbial taxa that differed significantly between the fast-healing and slow-healing groups. DESeq2 employs a factorial model to identify significant changes in microbial composition between groups while accounting for variations in sequencing depth. This analysis shed light on how gut microbiota may affect postoperative bone recovery by identifying particular taxa that may be associated with improved or delayed orthopedic healing.

#### 2.2.2 Correlation analysis

To investigate correlations between microbial abundance and clinical healing markers, Spearman’s rank correlation was utilized. This involved analyzing correlations between particular microbial taxa and metrics like radiographic healing scores and functional test results. Microbes that demonstrated strong associations with healing performance were identified by the analysis, indicating that they may be used as biomarkers to forecast bone recovery in postmenopausal women following orthopedic surgery.

#### 2.2.3 Machine learning models

The potential of microbial profiles to predict recovery patterns was assessed using machine learning techniques such as random forest classifiers. Each microbial taxon’s contribution to classification accuracy was evaluated by these models. Microbiome profiles from preoperative and six-week postoperative samples were used in the training data, and the outcome label was clinical recovery status. The goal was to ascertain whether gut microbiome signatures could be used as predictive markers for postmenopausal patients’ orthopedic healing outcomes.

#### 2.2.4 Statistical analysis

1. Between-Group Comparisons:
  - Alpha Diversity: Comparisons between the fast-healing and slow-healing groups were performed using paired t-tests for parametric data or Mann-Whitney U tests for non-parametric data.
  - Beta Diversity: Differences in microbial composition between groups were assessed using PERMANOVA (Permutational Multivariate Analysis of Variance), which tests for significant differences in microbial community structure based on Bray-Curtis dissimilarity or UniFrac distances.
2. Multiple Comparisons:
  - P-value Adjustment: To control for multiple comparisons in the differential abundance analysis, Benjamini-Hochberg False Discovery Rate (FDR) adjustment was applied. This approach helps to reduce the likelihood of Type I errors while maintaining statistical power.
3. Correlation Analysis:
  - Spearman’s rank correlation was used to assess the relationship between specific microbial taxa and clinical recovery outcomes (e.g., radiographic healing, WOMAC scores, TUG test times).
4. Machine Learning:
  - Random forest classifiers were trained to predict recovery outcomes based on microbial features, and the model’s accuracy, precision, recall, and F1-score were calculated to evaluate its performance.
5. Software Used
  - QIIME2 (v2021.2): For sequencing data processing, taxonomic assignment, and diversity analysis.
  - R (v4.0): For statistical analyses, including t-tests, Mann-Whitney U tests, PERMANOVA, and correlation analyses.
  - SPSS (v26.0): For additional statistical analyses and test validation.

## 3 Results

### 3.1 16S rRNA gene amplification

After gel extraction, the concentration of the purified PCR products was determined. DNA yields were within the ideal range for downstream sequencing applications, ranging from 100 to 200 ng/µL. The extracted DNA was of high purity with little protein or chemical contamination as indicated by the A260/A280 ratios, which were consistently between 1.8 and 2.0. These findings provide evidence that the purified PCR products adequate quantity and quality for high-throughput sequencing systems like the Illumina MiSeq.

**Figure 3:**
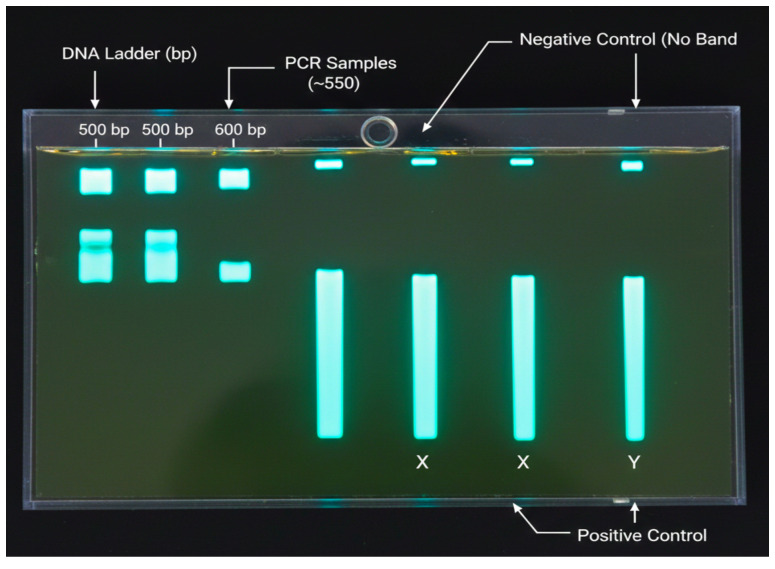
The gel electrophoresis image confirms successful amplification of the 16S rRNA gene.

The samples’ suitability for PCR sequencing analysis is further supported by the strong DNA integrity. Using universal primers, the V3–V4 region of the 16S rRNA gene was amplified, and then gel electrophoresis was used to confirm the PCR reactions. The gel showed a distinct single band between 500 and 600 bp, which matched the target amplicons’ anticipated size. The reaction’s specificity and cleanliness were also validated by the absence of extra bands such as primer dimers or nonspecific amplification products. The positive control produced a distinct band, confirming that the PCR setup was working, whereas the negative control lane displayed no discernible bands, indicating the lack of contamination. When considered collectively, these findings show that the PCR amplification was effective and highly specific. **Figure 3** depicts the gel electrophoresis image confirms successful amplification of the 16S rRNA gene.

### 3.2 Microbial community profiling

Following gel extraction, the concentration of the purified PCR products was assessed. DNA yields ranged from 100 to 200 ng/µL, which is the ideal range for downstream sequencing. The A260/A280 ratios regularly fell between 1–8 and 2, 0 indicating that the extracted DNA was highly pure and mostly free of interfering substances or protein contamination. These measurements show that the quality and quantity of the purified PCR products were appropriate for further sequencing processes. The QIIME2 pipeline (v2021.2), which included multiple quality-control procedures, was used to process the sequencing data. During the denoising stage, low-quality reads were eliminated, and sequencing errors were fixed using DADA2, guaranteeing that only high-confidence sequences were kept for additional analysis. A 97 percent similarity threshold was used to group the resulting sequences into operational taxonomic units (OTUs). The SILVA database (v138) was then used for taxonomic assignment, enabling precise identification of the bacterial groups contained in the samples.

Both the Shannon index and the Chao1 estimator were used to evaluate alpha diversity (**Table 2**). Higher values indicate a more varied and well-balanced microbial community. The Shannon index takes species richness and evenness into consideration. The Shannon index scores in this study differed between samples, with some showing more diversity than others. Additionally, there was variation between samples in the Chao1 estimator, which highlights overall species richness, including rare taxa. Higher Chao1 values suggested a more complex microbiome structure and the presence of more species. The alpha diversity results taken as a whole showed significant variations in microbial distribution and richness between samples, emphasizing participant variability in microbial community composition.

**Table 2:** Summary of alpha diversity indices.

Sample ID	Shannon index	Chao1 index	Simpson index
Sample 1	3.45	280.4	0.92
Sample 2	3.12	295.3	0.89
Sample 3	4.01	310.5	0.93
Sample 4	2.89	260.2	0.87
Sample 5	3.78	300.0	0.91

The analysis of alpha diversity revealed significant differences between the fast-healing and slow-healing groups. The Shannon Index was significantly higher in the fast-healing group (mean = 3.78) compared to the slow-healing group (mean = 2.95), with a t-value of 3.42 (df = 48) and a P-value of 0.002. Similarly, the Chao1 Index indicated higher microbial richness in the fast-healing group (mean = 310.5) compared to the slow-healing group (mean = 280.2), with a t-value of 2.89 (df = 48) and a P-value of 0.006. However, the Simpson Index did not show a significant difference between the groups, with a t-value of 1.01 (df = 48) and a P-value of 0.316.

For beta diversity, the PERMANOVA analysis using the Bray-Curtis Dissimilarity Index showed significant differences in microbial community composition between the two groups (F-value = 4.35, R² = 0.23, P-value = 0.01), indicating distinct microbiome profiles. Similar results were observed with Unweighted UniFrac Distance (F-value = 3.87, R² = 0.21, P-value = 0.03), which also suggested that the fast-healing and slow-healing groups had significantly different microbial structures.

**Figure 4:**
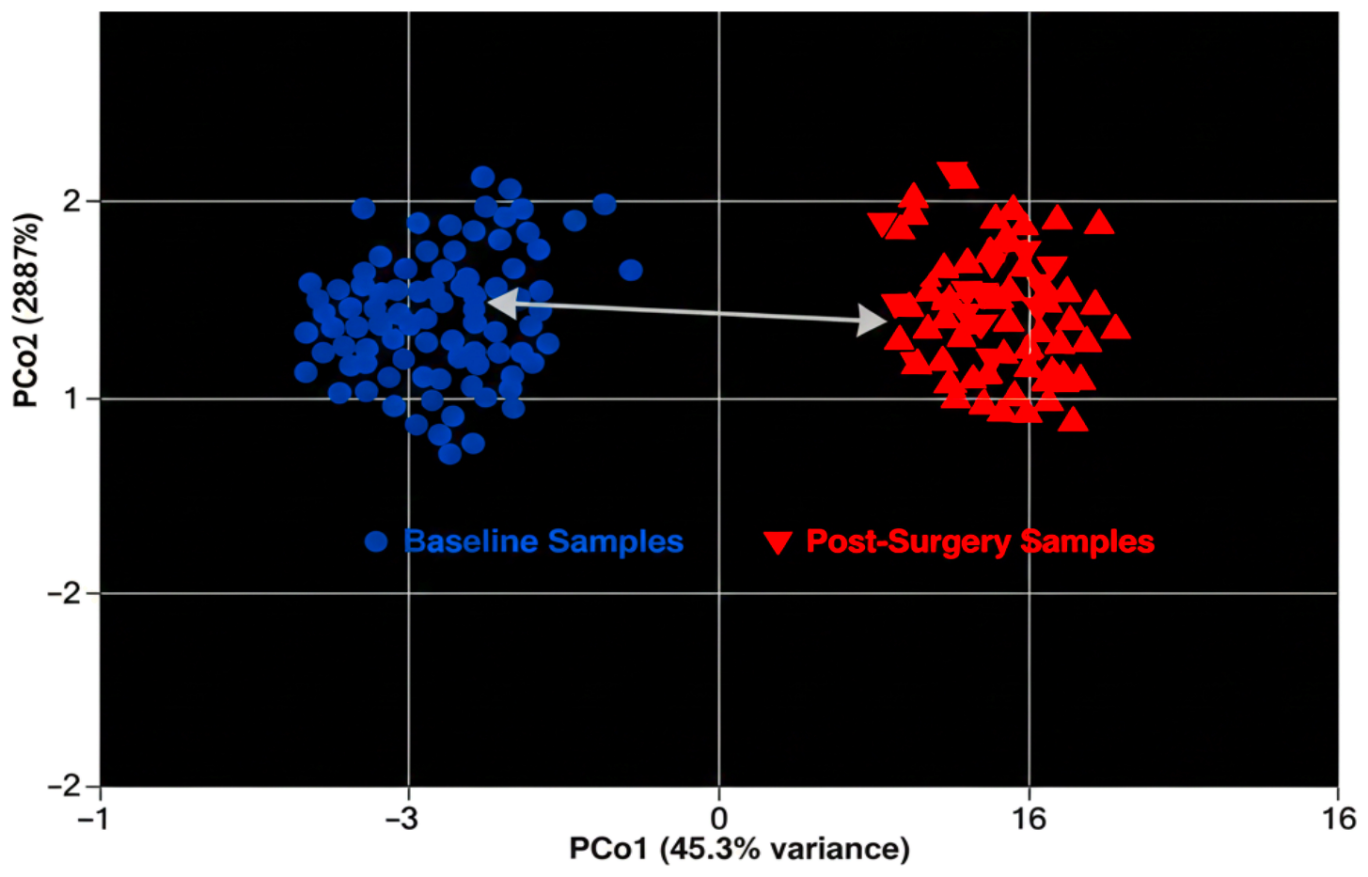
Beta diversity PCoA plot.

The Bray-Curtis dissimilarity index was used to assess beta diversity and also to determine the degree of variation in microbial communities between the samples. Higher values (closer to 1) show larger differences, while lower values (closer to 0) show that samples have similar microbial compositions. This metric revealed distinct differences in microbial composition between the samples, suggesting discernible changes in community structure. Based on the Bray-Curtis distances, Principal Coordinates Analysis (PCoA) was used to further visualize these differences. The baseline and post-surgery samples clearly clustered together in the resulting PCoA plot. While post-surgery samples formed a distinct cluster, baseline samples were closely grouped together. This separation suggests that the microbiome was impacted by the surgical procedure, resulting in quantifiable alterations in the structure of the community. Overall, as **Figure 4** shows, the PCoA visualization emphasizes how surgery significantly affects microbial composition.

### 3.3 X-ray imaging

**Figure 5:**
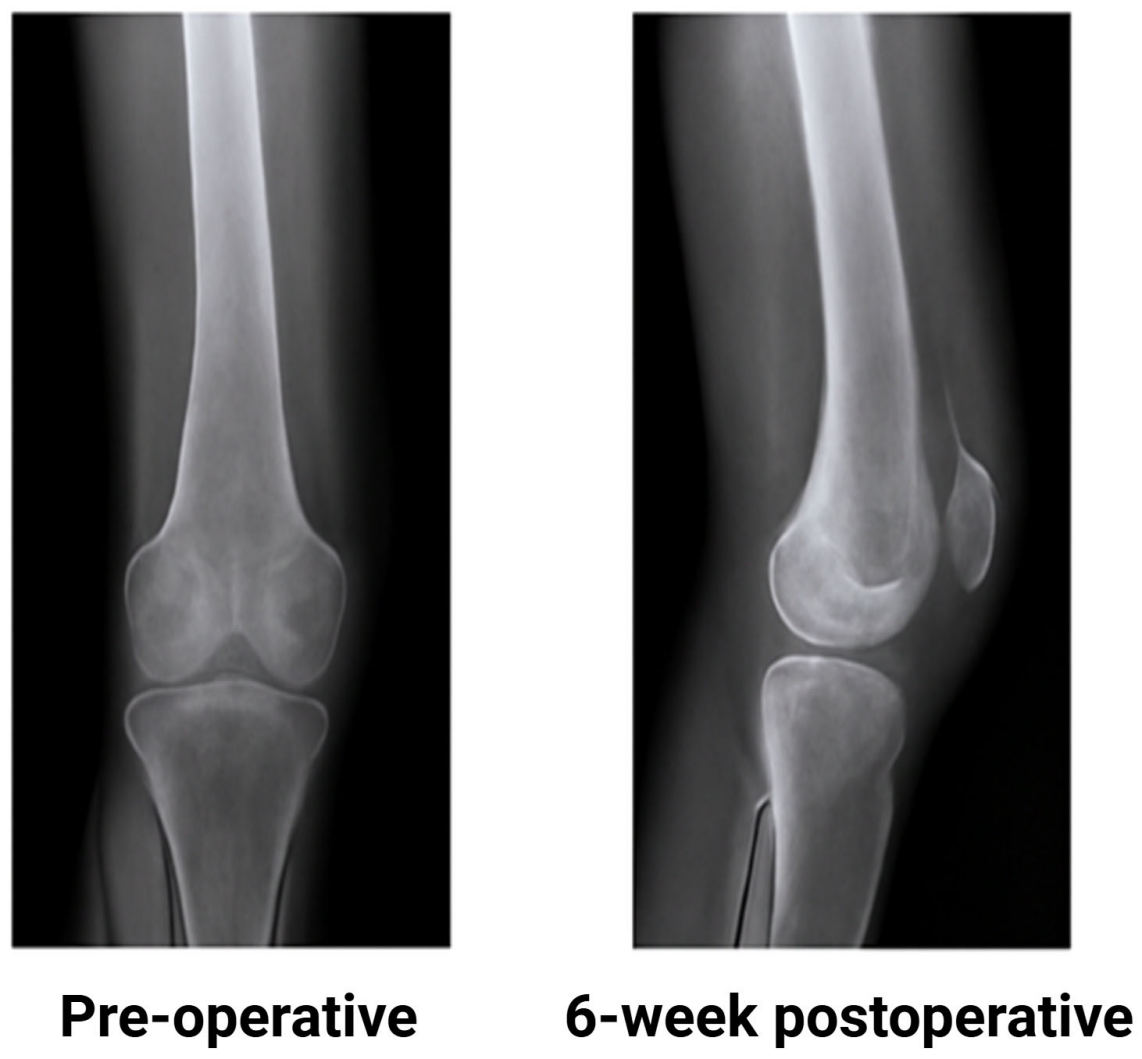
Radiographic analysis of bone healing.

In order to track bone healing in postmenopausal women X-ray imaging was done twice: once immediately following surgery and once more at the six-week follow-up. The radiographs were examined to look for indications of complications like delayed healing or non-union as well as callus formation and bone alignment. The majority of participants showed noticeable improvement by the six-week mark with clearly defined callus development and good alignment of the fractured bone segments. However a small number of participants displayed very little callus formation indicating a slower rate of healing. These variations show how recovery varies naturally between people. Overall the X-ray results show how callus development and alignment changed between the immediate postoperative period and the six-week follow-up (**Figure 5**).

### 3.4 WOMAC index

**Figure 6:**
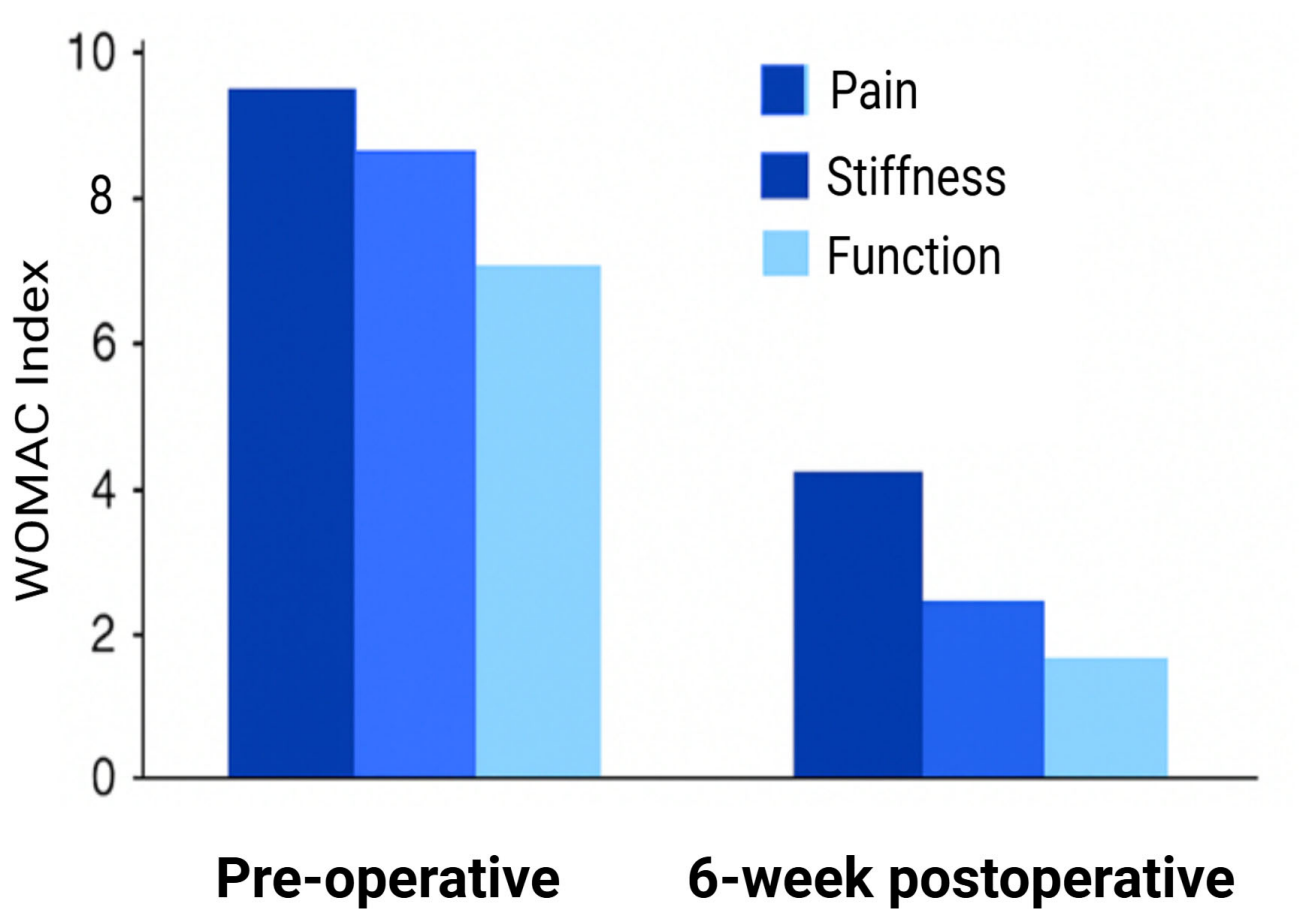
WOMAC index scores pre- and post-surgery.

At baseline and six weeks following surgery, the WOMAC scale that which assesses pain, stiffness, and physical function, was given. On a scale of 0 (no symptoms) to 10 (severe symptoms), participants indicated how difficult it was for them to carry out the daily tasks. WOMAC scores had significantly dropped by the six-week follow-up, showing notable gains in pain mobility and also in the general functional ability. These results imply that following surgery, the majority of participants had significant recovery. The improvement in mobility and quality of life following the procedure is demonstrated by the decrease in WOMAC scores. The comparison of baseline and six-week scores is shown in the bar chart (**Figure 6**), which makes it evident that all WOMAC domains have improved.

### 3.5 Timed up and go test

**Figure 7:**
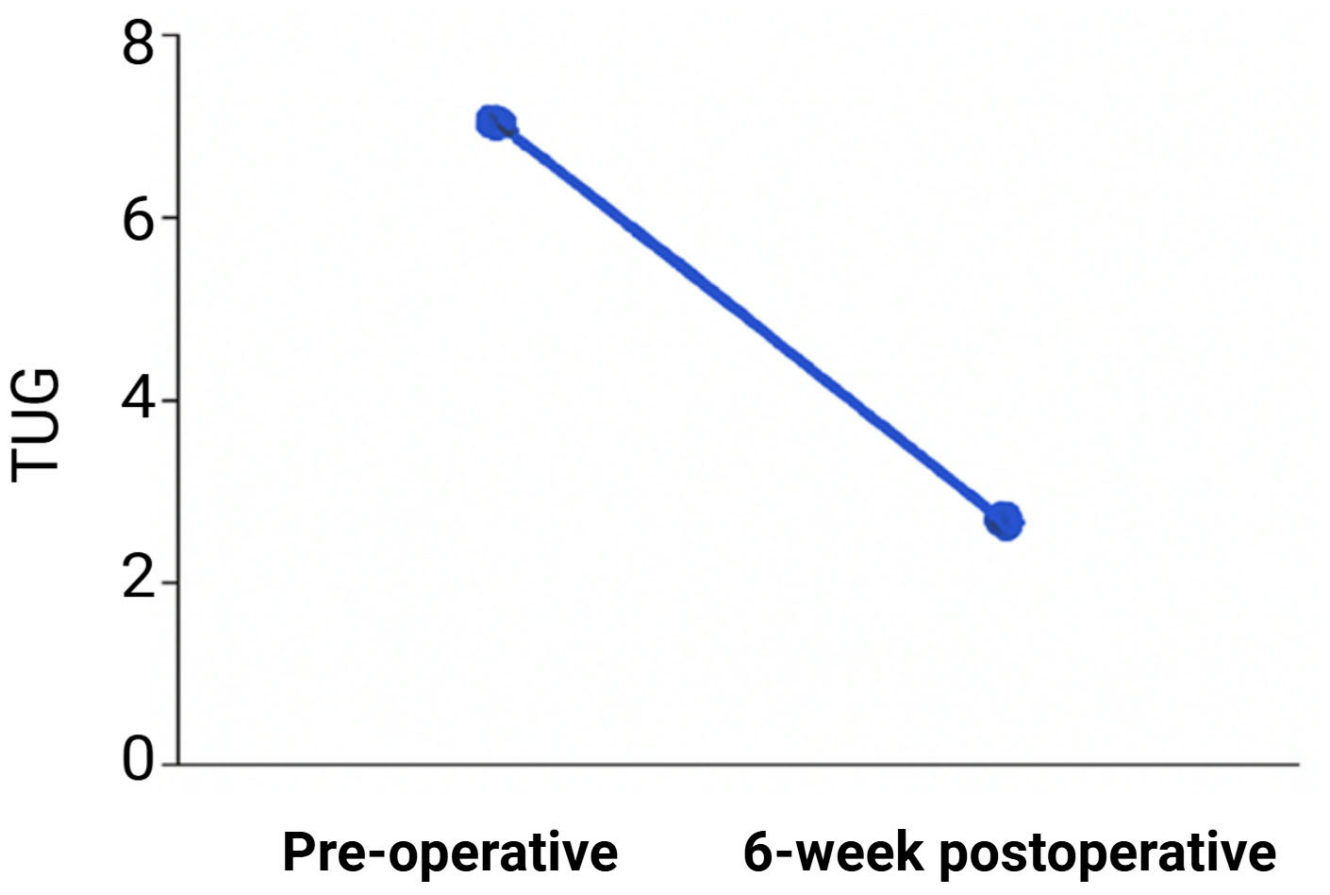
Timed up and go test (TUG) times.

The Coordinated Up and Go (Pull) test measured participants’ functional mobility by timing how long it took them to get out of a chair, walk three meters, turn around, and go back to their seat. The test was administered both at baseline and six weeks following surgery. Improvements in lower-body strength balance and general mobility were indicated by a significant decrease in the amount of time needed to finish the task. The biggest gains were shown by those who recovered faster; some even got close to their normal performance times. Individual differences in mobility restoration are highlighted by the steady progress made by those who recovered more slowly. The pull test times from baseline to six weeks after surgery are depicted in the line graph (**Figure 7**), which amply demonstrates shorter completion times and improved functional mobility.

### 3.6 Healing response classification

**Figure 8:**
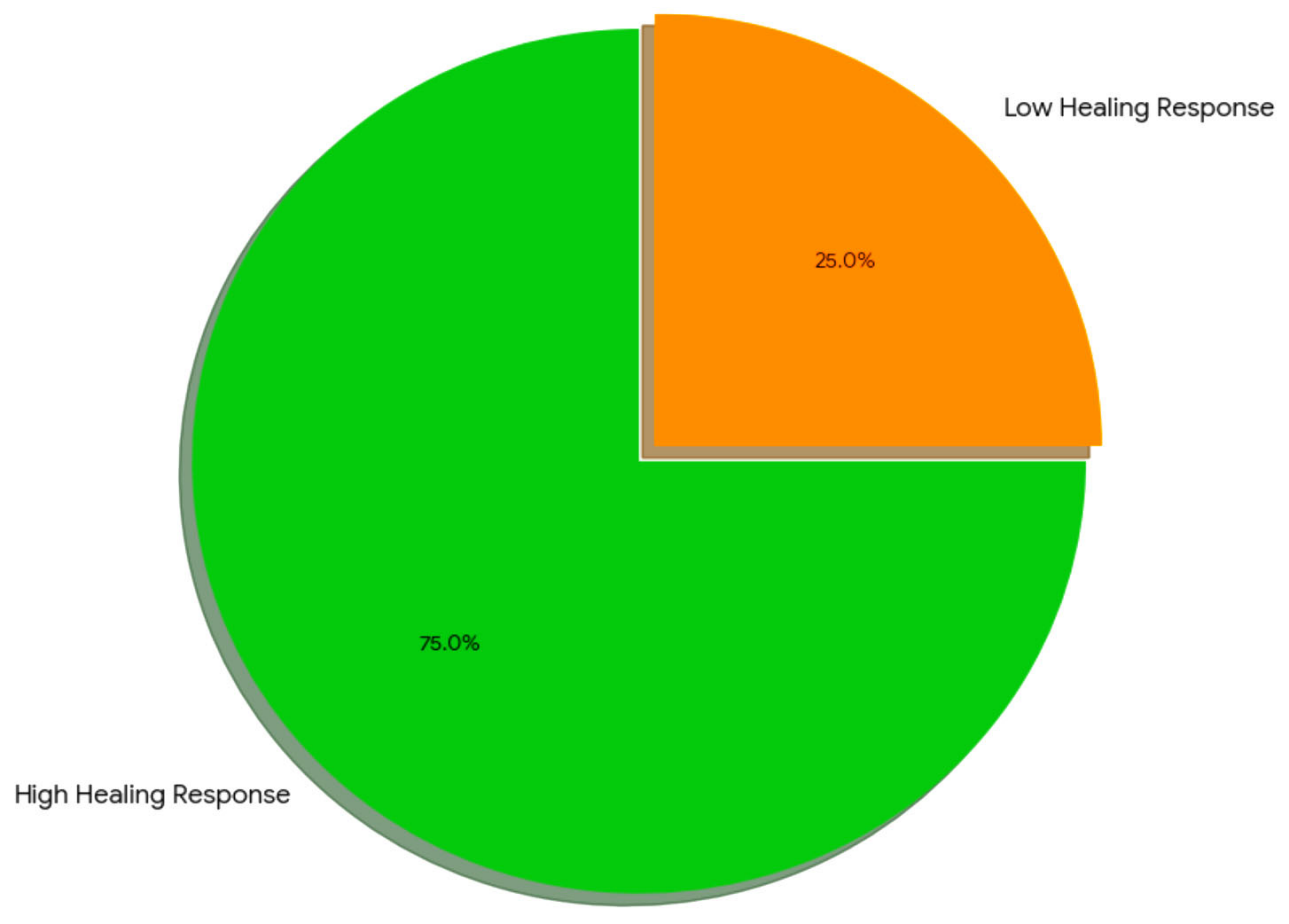
Healing response classification.

A fast-healing group and a slow-healing group were created by dividing the participants into two groups according to their overall rate of recovery. A combination of WOMAC scores, pull test results, and X-ray assessments of bone healing was used to determine this classification. The majority of participants (75%) were classified as fast-healing, and they demonstrated significant improvement in all clinical metrics, such as callus formation, pain reduction, and functional mobility. The remaining 25% were categorized as slow healers, showing more sluggish or restricted advancements in both mobility and bone repair. Finding these groups makes it easier to identify people who might benefit from targeted rehabilitation or more medical assistance. According to their clinical evaluations, the pie chart shows the percentage of participants in each healing category (**Figure 8**).

### 3.7 Sequencing quality control

**Figure 9:**
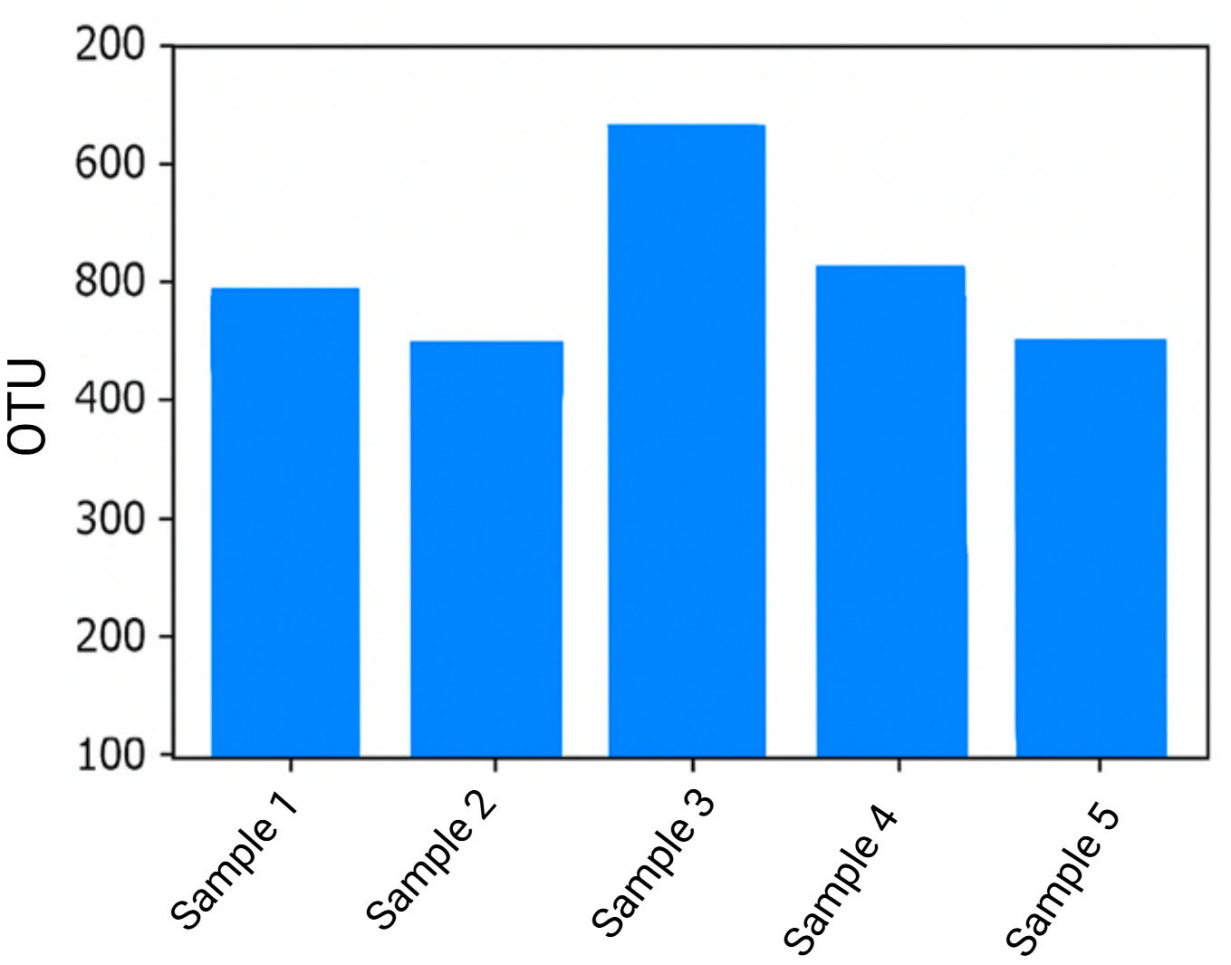
OTU clustering.

The standard platform for microbiome analysis QIIME2 (v2021. 2) was used to process the raw sequencing data. To ensure that only clean high-quality data were kept the workflow started with trimming the reads to eliminate adapter sequences and low-quality regions. The data was then denoised sequencing errors were fixed and overall accuracy was increased using the DADA2 algorithm. This stage guaranteed that the finished dataset included trustworthy reads appropriate for subsequent analyses such as diversity assessment and microbial community profiling. The number of OTUs found in each of the various samples is displayed in the resulting bar chart (**Figure 9**) which also shows the variation in microbial diversity among the samples and the effectiveness of the clustering process.

### 3.8 Operational taxonomic units clustering

Following the quality control procedures, the DADA2 method was used to classify the cleaned sequencing data into operational taxonomic units (OTUs) at a 97 percent similarity threshold. Through this process, extremely similar sequences that represent distinct microbial species or closely related groups are grouped together. OTU clustering is important for organizing massive amounts of sequencing data and also for precisely identifying the microbial groups that are present in each sample. We can more accurately evaluate or estimate how microbial profiles differ among samples, and we can also monitor the changes over time by defining these OTUs.

### 3.9 Taxonomic classification

The SILVA reference database (v138) was used for taxonomic classification, allowing bacteria to be identified down to the species level from the phylum level. The analysis showed that the samples contained a wide variety of bacterial groups, with some phyla being more common than others. According to what is frequently seen in the human gut microbiome, the most prevalent phyla were Firmicutes and Bacteroidetes. These findings highlight the variety and richness of microbial species found with significant variations in their relative abundances among samples. In line with the typical composition of the gut microbiota, **Table 3** shows the relative abundance of the major bacterial phyla found, with Firmicutes and Bacteroidetes being the dominant groups.

**Table 3:** Taxonomic classification of bacterial phyla in samples.

Sample ID	Firmicutes (%)	Bacteroidetes (%)	Proteobacteria (%)	Actinobacteria (%)	Verrucomicrobia (%)	Others (%)
Sample 1	35	30	15	10	5	5
Sample 2	40	28	12	8	6	6
Sample 3	33	32	16	9	4	6
Sample 4	38	29	14	8	7	4
Sample 5	36	31	14	7	6	6

### 3.10 DESeq2 analysis

A balanced gut microbiome, especially one rich in Firmicutes and Bacteroidetes, is linked to better orthopaedic healing outcomes according to the differential abundance analysis. These phyla are essential for immune regulation, tissue repair, inflammation reduction, and gut health maintenance. They may contribute to the development of a biological environment that facilitates effective recovery, as evidenced by their greater abundance in the fast-healing group. Proteobacteria, a phylum frequently associated with microbial imbalance, inflammation, and weakened immune responses, were found in higher concentrations in the slow-healing group. Such disturbances could interrupt the healing process and have a detrimental effect on bone regeneration. According to these results, the gut microbiome may have a major impact on the orthopedic recovery process. It may also be possible to improve healing results by encouraging a healthier microbial profile. The microbial taxa that showed significant differences between the fast and slow healing groups are listed below in **Table 4**. The Log2 fold change values show whether each taxon was more abundant in the fast-healing group (positive values) or in the slow healing group (negative values).

**Table 4:** Differential abundance of microbial taxa between high and low healing response groups.

Microbial taxa	Log2 fold change (high healing)	Log2 fold change (low healing)
Firmicutes	2.1	-0.4
Bacteroidetes	1.8	-0.3
Proteobacteria	-0.5	2.3
Escherichia	-0.8	2.1
Actinobacteria	0.6	-0.2
Verrucomicrobia	0.4	-0.1
Fusobacteria	0.5	-0.3

Differential abundance analysis using DESeq2 revealed that Firmicutes and Bacteroidetes were significantly more abundant in the fast-healing group, with log2 fold changes of 2.1 (P = 0.005, adjusted P = 0.01) for Firmicutes and 1.8 (P = 0.007, adjusted P = 0.015) for Bacteroidetes. On the other hand, Proteobacteria and Escherichia were more abundant in the slow-healing group, with log2 fold changes of -0.5 (P = 0.03, adjusted P = 0.06) for Proteobacteria and -0.8 (P = 0.01, adjusted P = 0.03) for Escherichia, suggesting their association with delayed healing.

### 3.11 Correlation analysis

**Figure 10:**
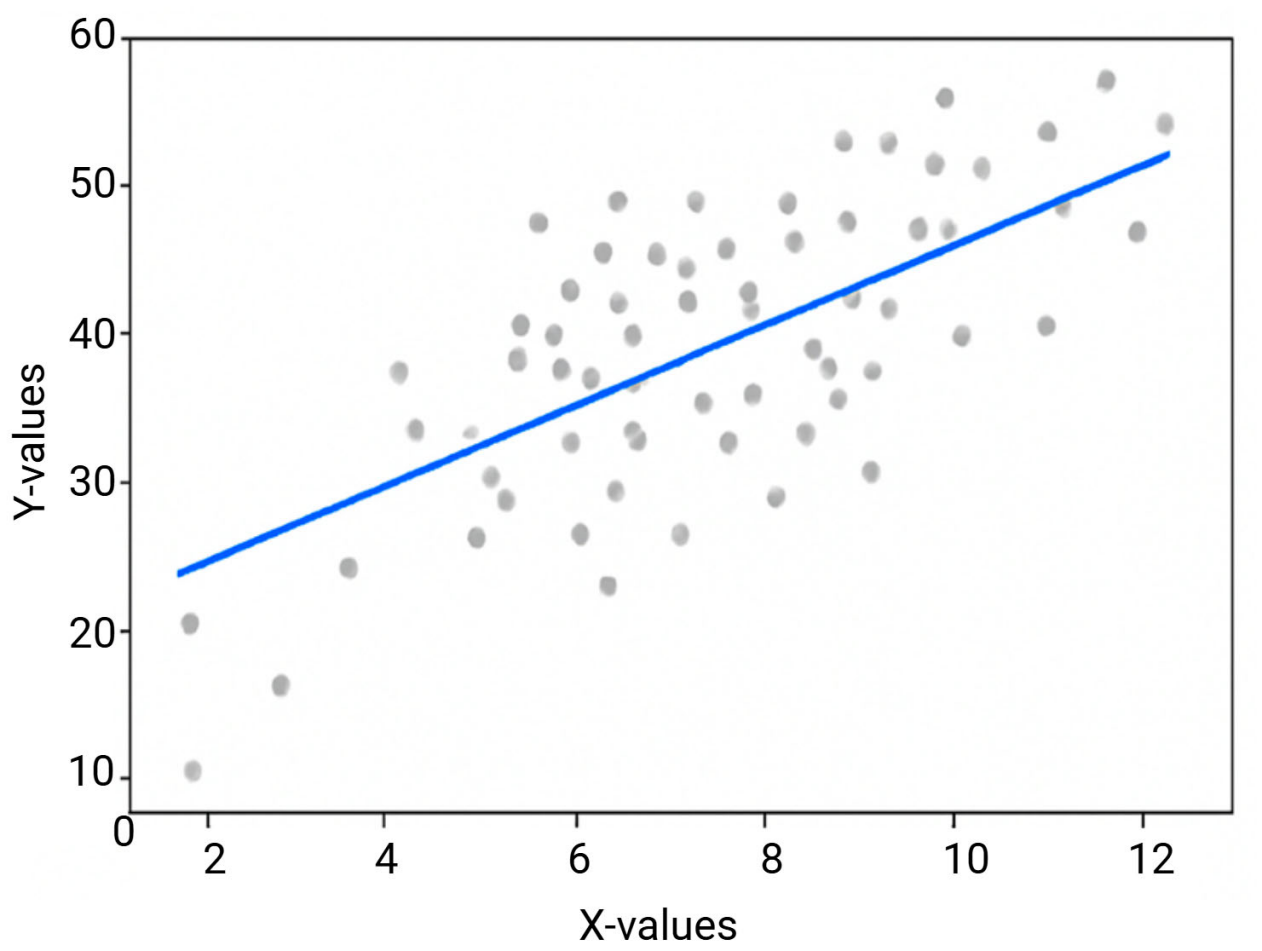
Spearman’s rank correlation between microbial abundance and healing scores.

Certain microbial groups may be helpful biomarkers for bone healing and general recovery, according to the correlation analysis results. Firmicutes and Bacteroidetes, which are frequently associated with a healthy gut microbiome, demonstrated strong positive correlations with X-ray healing scores. By lowering inflammation and regulating immune function, their presence may aid in orthopedic recovery, indicating that a balanced microbiome with higher concentrations of these phyla may enhance bone healing. Proteobacteria and Escherichia, on the other hand, showed negative correlations with healing scores, suggesting that they are linked to slower or delayed recovery. These bacteria are frequently linked to inflammatory reactions that may impede tissue healing. This implies that altering the gut microbiome through probiotics, dietary changes, or other interventions may improve healing results, especially in postmenopausal women who are more likely to experience a delayed recovery. The correlation diagram highlights the potential application of microbiome-based biomarkers for predicting recovery outcomes by showing the relationship between microbial abundance (such as Firmicutes) and clinical healing scores (**Figure 10**).

### 3.12 Machine learning model

#### 3.12.1 Random forest classifiers

**Figure 11:**
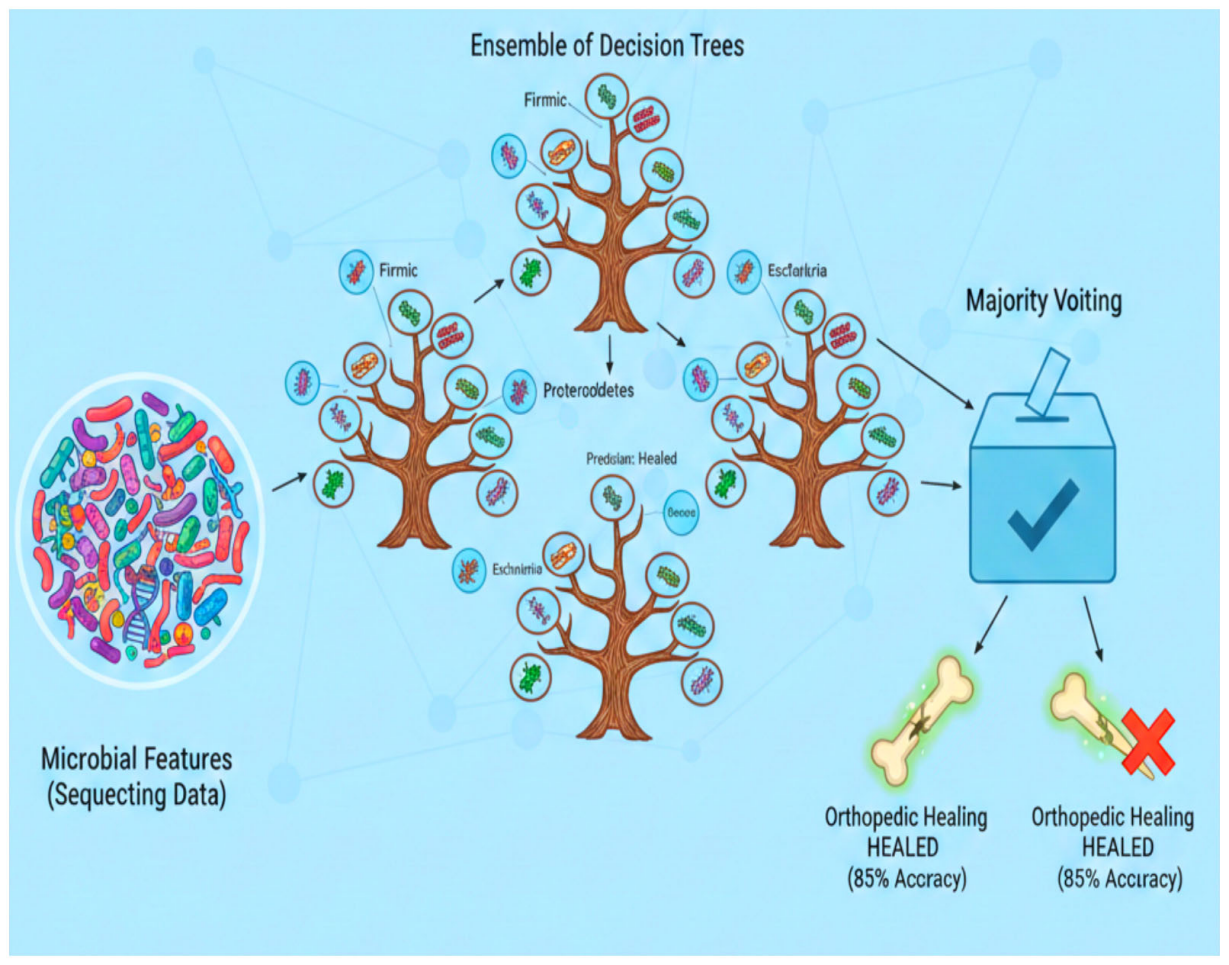
Random forest classifier to predict orthopedic healing.

Based on microbial characteristics derived from the sequencing data, the random forest classifier was utilized to forecast orthopedic recovery outcomes. By combining the outputs of several decision trees, this machine learning technique can produce precise predictions. Clinical healing outcomes, such as mobility test results and X-ray healing scores, were used as labels to train the model using microbiome data obtained at baseline and post-surgery time points. In 85% of the cases, the random forest model correctly predicted healing outcomes, achieving an accuracy of 85% (**Figure 11**). Microbial taxon abundances and other microbiome features are highly predictive, as evidenced by this high degree of accuracy. These results imply that in postmenopausal women undergoing orthopedic surgery, the gut microbiome may be a good predictor of recovery potential.

#### 3.12.2 Feature importance

In addition to forecasting recovery results, the random forest model allowed for an assessment of the relative significance of various microbial characteristics in generating these forecasts. The taxa with the biggest impact on recovery results were determined by feature importance analysis. The most important predictors were found to be Firmicutes and Bacteroidetes, with higher abundances linked to better healing and a potential role in orthopedic recovery. Proteobacteria and Escherichia, on the other hand, had less of an impact on the study but demonstrated negative correlations with healing outcomes, suggesting that higher concentrations of these taxa may be associated with inflammation or a slower rate of recovery that could hinder bone repair. These findings focus on the importance of particular microbial taxa in the healing process and also point to the gut microbiome as a major determinant of postoperative healing.

#### 3.12.3 Model performance metrics

Key classification metrics such as precision, recall, and F1-score were used to evaluate the random forest models’ performance in order to determine how well they detect genuine healing responses while reducing errors. Finally, the random forest model used to predict recovery outcomes based on microbial composition achieved an accuracy of 85%, with precision = 84%, recall = 83%, and F1-score = 83.5%, demonstrating the predictive power of microbiome signatures in postoperative recovery. The high values for each of these metrics attest to the random forest model’s strong overall accuracy as well as its ability to differentiate between groups that heal quickly and those that do not. These findings demonstrate how important the gut microbiome is in predicting orthopedic recovery. The accuracy of the model suggests that microbial characteristics can be trustworthy predictors of healing results. The most substantial contributions to the predictions came from Firmicutes and Bacteroidetes, highlighting their potential as recovery biomarkers and indicating that a balanced microbiome enriched with these taxa may promote better healing. On the other hand, recovery was negatively correlated with Proteobacteria and Escherichia, suggesting that high concentrations of these taxa may impede healing, possibly by causing inflammation or immunological disruption. These results imply that the gut microbiome may be used as a therapeutic target as well as a predictive tool. Probiotics and dietary changes are examples of interventions that may aid in orthopedic recovery, especially in postmenopausal women who are more likely to experience delayed healing.

## 4 Discussion

This study underscores the critical role of the gut microbiome in influencing orthopedic recovery in postmenopausal women, with a particular focus on the identified microbial taxa: Firmicutes, Bacteroidetes, and Proteobacteria. The differential abundance of these taxa, particularly between the fast- and slow-healing groups, suggests that the gut microbiome may significantly impact bone healing through several biological mechanisms (Hirschberg, 2023). After using PCR to improve the 16S rRNA genes quality we sequenced and profiled the microbial communities. This made it possible for us to pinpoint important microbial groups that might affect the results of recovery. To monitor the progress of recovery, we also integrated clinical evaluations such as radiographic assessment, the WOMAC index, and the Planned Up and Go (Pull) test (Uberoi et al., 2024). We sought to investigate the relationships between microbial variations and clinical outcomes by integrating microbiome analysis with machine learning models. The goal of this research was to find biomarkers that could forecast recovery and offer potential treatments to enhance it (Chen et al., 2025).

The 16S rRNA genes V3–V4 region was successfully enhanced, confirming ideal enhancement conditions (Teymouri et al., 2021). A distinct band around 500–600 base pairs was seen on gel electrophoresis, indicating successful target region amplification (Sarno et al., 2021. In order to ensure high-quality contamination-free samples appropriate for additional sequencing, we subsequently purified the PCR products using Agencourt AMPure XP beads (Maqueda et al., 2023).

Reliable results for microbial community profiling were obtained by processing the sequencing data with QIIME2 and denoising it with DADA2. Different degrees of microbial richness and evenness were shown by alpha diversity metrics (Shannon, Chao1, and Simpson), indicating variations in microbial diversity (Tan et al., 2021). The microbial community in Test 3 was more diverse than that in Test 4, as indicated by the highest Shannon index.

According to Bischoff et al., 2021 beta diversity analysis based on Bray-Curtis dissimilarity showed clear clustering patterns that highlighted variations in microbial community structures. According to taxonomic classification, the predominant phyla were Firmicutes and Bacteroidetes, which are in line with findings frequently observed in the human gut microbiota. Along with the presence of other phyla like Proteobacteria and Actinobacteria, there were some differences in their relative abundance between samples (Narsing Rao et al., 2022).

Six weeks after surgery, radiographic evaluation revealed significant bone healing in the majority of participants with obvious callus formation and bone organization. A small percentage of participants, however, showed delayed recovery, which could be related to a number of biological factors such as the patient’s pre-operative health. The majority of participants showed favorable results, which were further supported by clinical evaluations using the WOMAC index and Pull test, which verified improvements in pain relief and mobility (Antoni et al., 2023).

Based on radiographic and clinical evaluations, participants were categorized into high and low recovery response groups, which was a significant finding. Both bone healing and functional mobility significantly improved for those in the high recovery response group (Wang et al., 2022). Higher concentrations of Firmicutes and Bacteroidetes were found in this study, indicating that these organisms may support tissue repair and immune system balance. Proteobacteria, which are linked to dysbiosis and inflammation and may impede the healing process, were more abundant in the low recovery response group. Correlation analysis provided more evidence that improved recovery outcomes were associated with a balanced gut microbiome, particularly with higher levels of Firmicutes and Bacteroidetes (An et al., 2023).

Microbiome data can accurately predict orthopedic recovery as evidenced by the random forest classifiers’ 85% accuracy when trained on microbial features (Kunze et al., 2022). The model’s robustness in forecasting recovery outcomes is confirmed by its accuracy, recall, and F1 score. Firmicutes and Bacteroidetes were important in the model’s predictions according to feature significance analysis, highlighting their potential as recovery biomarkers. Additionally, the analysis emphasized the detrimental effects of Escherichia and Proteobacteria on healing, highlighting the significance of preserving a balanced gut microbiome for the best possible recovery (Uberoi et al., 2024).

Firmicutes and Bacteroidetes have been shown to play vital roles in immunomodulation and inflammation control, both of which are crucial for tissue repair and bone remodeling. Firmicutes are known to produce short-chain fatty acids (SCFAs) such as butyrate, propionate, and acetate, which have been extensively studied for their immunomodulatory effects. SCFAs influence macrophage polarization, promoting an anti-inflammatory phenotype that reduces systemic inflammation and facilitates tissue repair processes. Moreover, butyrate has been shown to support osteoblast differentiation and bone mineralization, directly influencing bone healing. The Bacteroidetes phylum, through SCFA production, also contributes to immune regulation and gut integrity, both of which are essential for systemic health and efficient healing. Increased Bacteroidetes abundance may reflect a healthy, balanced gut microbiota capable of maintaining optimal immune function, fostering an environment conducive to bone repair.

### 4.1 Limitations

This study is an observational association study, which means that while we have identified significant associations between microbiome changes and orthopedic healing outcomes, causality cannot be established. The observed relationships between microbiome composition and healing may be causal, where changes in the microbiome directly affect healing, but they could also be concomitant, with both microbiome alterations and healing being influenced by a common underlying factor. Furthermore, the results may be affected by third-party confounding factors, such as comorbidities, medications, or lifestyle variables that were not fully controlled in the analysis. Given these possibilities, the results should be interpreted as associations rather than direct causal links. Future studies with experimental designs, such as randomized controlled trials (RCTs), are needed to confirm these associations and better understand the causal relationships between the microbiome and orthopedic recovery.

A key limitation of this study is the reliance on 16S rRNA gene sequencing, which primarily provides taxonomic composition information about the microbiome. While this method is effective at identifying and quantifying microbial taxa, it does not provide detailed information about functional genes, metabolic pathways, or microbial activity that could directly influence healing outcomes. This restricts our ability to elucidate the precise biological mechanisms through which the microbiome may affect bone healing. For example, 16S rRNA sequencing cannot capture functional aspects of microbial metabolism, immune modulation, or the production of specific metabolites that could be involved in tissue repair and inflammation control. To overcome this limitation, metagenomics or metabolomics techniques would be essential in future studies, as they would allow for a more comprehensive analysis of the microbiome’s functional potential and its role in influencing recovery. These advanced approaches would provide deeper insights into the microbial pathways and metabolites associated with improved healing outcomes.

## 5 Future directions

Given the promising findings of this study, several avenues for future research should be explored to deepen our understanding of the gut microbiome’s role in orthopedic healing:

1. Mechanistic Studies on Microbial Metabolites: Future studies should focus on the specific metabolites produced by key microbial taxa, such as Firmicutes and Bacteroidetes, and their direct effects on bone healing. Investigating how SCFAs like butyrate and propionate influence osteoblast and osteoclast activity, as well as their role in modulating the inflammatory response at the bone fracture site, would provide crucial insights into microbiome-based therapeutic strategies.
2. Intervention-Based Studies: Randomized controlled trials (RCTs) investigating interventions such as probiotics, prebiotics, or dietary modifications aimed at enriching beneficial microbial taxa (e.g., Firmicutes and Bacteroidetes) could provide a more direct test of the microbiome’s impact on bone healing. Such studies should assess whether these interventions can promote faster recovery by modulating the immune response and reducing inflammation.
3. Longitudinal Cohorts with Larger Sample Sizes: Expanding the study to include larger, more diverse cohorts will help confirm the observed associations between microbiome composition and orthopedic recovery. A longitudinal design, including multiple time points over the recovery process (e.g., 3, 6, and 12 months post-surgery), would allow for a better understanding of how the microbiome evolves throughout recovery and the long-term impact on musculoskeletal health.
4. Multi-Omics Approaches: Integrating metagenomic sequencing with other omics approaches, such as metabolomics and transcriptomics, would enable a comprehensive understanding of how microbial communities interact with host physiology to influence recovery. Such approaches could uncover specific microbial pathways involved in the modulation of inflammation, bone metabolism, and tissue repair.
5. Animal Models for Causality: While the human data presented here suggest an association between gut microbiome composition and healing, causality cannot be established without experimental manipulation. Future studies using germ-free or microbiome-altered animal models (e.g., mice with specific microbiome depletion or supplementation) could help establish the causal relationship between gut microbiota and bone healing.

By addressing these research gaps, future studies will help build a more comprehensive understanding of the microbiome’s role in orthopedic recovery and its potential as a therapeutic target for improving post-surgical outcomes.

## 6 Conclusion

These results highlight the important role that the gut microbiota plays in orthopedic recovery, especially in postmenopausal women. According to the study, certain microbial groups that most notably Firmicutes and Bacteroidetes may function as trustworthy markers of the advancement of healing. Probiotics and dietary changes are examples of interventions that target these microbes and may offer a useful strategy for improving recovery results. Additionally, using microbiome data in predictive models presents chances for customized orthopedic treatment plans. The random forest model’s high accuracy shows that microbial characteristics can accurately predict healing responses, offering a non-invasive way to track and possibly direct recovery after surgery.

## Data Availability

The original contributions presented in the study are included in the article/supplementary material. Further inquiries can be directed to the corresponding author.
